# Patterns and Correlates of Bone Mineral Density Parameters Measured Using Calcaneus Quantitative Ultrasound in Chinese Adults

**DOI:** 10.3390/nu17050865

**Published:** 2025-02-28

**Authors:** Peng Peng, Charlotte Clarke, Andri Iona, Neil Wright, Pang Yao, Yiping Chen, Dan Schmidt, Ling Yang, Dianjianyi Sun, Rebecca Stevens, Pei Pei, Xin Xu, Canqing Yu, Junshi Chen, Jun Lv, Liming Li, Zhengming Chen, Huaidong Du

**Affiliations:** 1 Medical Image Center, University Hospital Macau, Macau University of Science and Technology, Macau, China; 2 Clinical Trial Service Unit and Epidemiological Studies Unit, Nuffield Department of Population Health, University of Oxford, Oxford OX3 7LF, UK; 3 Department of Epidemiology and Biostatistics, School of Public Health, Peking University Health Science Center, Beijing 100083, China; 4 Peking University Center for Public Health and Epidemic Preparedness and Response, Beijing 100191, China; 5 Key Laboratory of Epidemiology of Major, Peking University, Ministry of Education, Beijing 100191, China; 6 Liuyang CDC, Liuyang 410300, China; 7 China National Center for Food Safety Risk Assessment, Beijing 100022, China

**Keywords:** bone mineral density (BMD), quantitative ultrasound (QUS), stiffness index (SI), T-score, Chinese

## Abstract

**Background:** Monitoring bone mineral density (BMD) in adults is critical for early detection of osteoporosis and prevention of fracture, for which quantitative ultrasound (QUS) is a good non-invasive tool. We examined the associations of QUS measures, including stiffness index (SI) and T-score, with socio-demographic, lifestyle, and anthropometric correlates and risk of subsequent fracture. **Methods**: Calcaneal QUS was performed using GE’s Lunar Achilles EXPII among 24,651 adults (mean age 59.5 years, 61.7% women) from the China Kadoorie Biobank study. Socio-demographic and lifestyle information was collected using an interviewer-administered electronic questionnaire, and anthropometrics were measured following standard protocols. Incidence of fracture and osteoporosis was recorded via linkage with nationwide health insurance database. Linear and Cox regression analyses were conducted, adjusting for potential confounders. **Results**: On average, men had higher SI (92.8 vs. 86.0) but lower T-score (−0.85 vs. −0.64) than women. In both men and women, advanced age and smoking were inversely associated with SI and T-score, while physical activity and tea drinking were positively so (*p* < 0.0001 for all). Except for height, all other anthropometric measures were significantly and positively associated with both BMD measures. With each SD lower SI, the risk of subsequent fracture was 26% (95% confidence interval: 10–44%) and 40% (25–57%) higher in men and women, and the corresponding associations of T-score were identical. **Conclusions**: Among Chinese adults, the SI and T-score provided by Achilles EXPII had similar patterns and predictive values for subsequent fracture, despite the T-score for men and women not being directly comparable because of gender-specific references used. Future studies are needed to confirm or refute the causality of relationship between lifestyle and anthropometric factors and BMD.

## 1. Introduction

Osteoporosis is a common metabolic disease characterised by reduced bone mass and microarchitectural deterioration of bone tissue, with an increased risk of fracture [1]. Worldwide, osteoporosis causes about 9 million fractures annually. According to the World Health Organisation (WHO), one in three women and one in five men over the age of 50 experience osteoporotic fracture, which causes an annual global loss of 5.8 million healthy life years to disability [2]. With population aging and global urbanisation, the incidence of this disease is expected to increase substantially, having significant social and economic implications [3]. Monitoring bone mineral density (BMD) among older adults is critical for early detection of osteoporosis and prevention of fracture, for which dual-energy X-ray absorptiometry (DXA), particularly when performed at the central skeleton sites like the spine and hip (i.e., central BMD), is one of the gold standard methods [4]. However, it may not be a feasible choice for large-scale community-based epidemiological studies due to the cost and size of devices and the potential risk related to the ionising radiation [5]. Quantitative ultrasound (QUS) is a good non-invasive alternative tool for assessment of BMD and predicting fracture risk in large populations [6,7], particularly in less developed low- and middle-income countries, such as China.

The China Kadoorie Biobank (CKB) study is a large-ongoing prospective study including 0.5 million adults recruited from 10 geographically defined locations in China during 2004–2008 [8]. In 2013–2014, a resurvey of ~5% randomly selected surviving participants of the CKB (~25,000) was conducted, during which calcaneus QUS scan was performed to assess BMD [9]. The device uses low-frequency ultrasonic waves to measure speed of sound (SOS, expressed as m/s) and broadband ultrasound attenuation (BUA, expressed as dH/MHz) and combine them to form a clinical measure called the stiffness index (SI, expressed as a percentage of the result from a healthy reference), which could be considered as an indirect representation of microarchitectural property of bone and a surrogate parameter for BMD assessed by DXA [10,11]. SI was then used to derive the T-score through comparing individual SI measures to the gender-matched young normal reference mean on a standard deviation scale [12].

Besides a few well-established determinants for osteoporosis, such as aging, female sex, and physical inactivity, several risk factors for poor bone health have been identified, including body composition indices and lifestyle factors. However, such associations with SI have not yet been established, particularly in non-Western populations. We therefore aimed to study the patterns as well as lifestyle and anthropometric correlates of BMD parameters (i.e., SI and T-score) in this large group of Chinese men and women aged 40 years and older from the CKB, as well as to investigate and compare predictive values of different BMD parameters for subsequent incidence of fractures. The information would be greatly valuable for understanding the age-, gender-, and other subgroup-specific risks of fracture in the population and the usefulness of QUS measures in assessing osteoporosis risk.

## 2. Materials and Methods

### 2.1. Study Population

The CKB study is an ongoing prospective cohort study of 0.5 million Chinese adults, aged 30–79 years at baseline, recruited from 10 geographically diverse regional sites that were chosen according to local disease patterns, exposure to certain risk factors, population stability, quality of death and disease registries, and local commitment and capacity [8,13]. In 2013–2014, a random sample of surviving participants (~34,000) was invited to participate in the resurvey to help assess changes of baseline exposures over time and to collect new data on emerging risk factors for chronic diseases. About ~75% (n = 25,239) of the invitees participated in the resurvey. They were interviewed using a laptop-based interviewer-administered questionnaire (similar to the CKB baseline questionnaire and see https://www.ckbiobank.org/files/qs_2ndresurvey_11dec2013.pdf (accessed on 24 February 2025)) and were measured for a range of parameters including calcaneus QUS scan. Ethics approval was obtained from the University of Oxford Tropical Research Ethics Committee (OXTREC), the Chinese Academy of Medical Sciences, and all regional sites. All participants provided written informed consent.

### 2.2. Calcaneus QUS Scan

For each participant, right and left calcaneal QUS were performed by trained staff members using Lunar Achilles EXPII (GE healthcare, Chicago, IL, USA), which is a US FDA-approved QUS scanner with a temperature-controlled water-based system used as a coupling medium for wave propagation. This device measures BUA and SOS and calculates SI according to the following equation: SI = (0.67 × BUA + 0.28 × SOS) – 420 [14]. According to the manufacturer, the SI is less dependent on the temperature of the measurement environment than BUA or SOS alone (because BUA and SOS vary in the opposite direction with temperature). T-score was also provided by the device through comparing the SI with imbedded reference databases (Supplementary Table S1). We followed the standard operating procedures provided by the manufacturer, and a reference database for adult Chinese was chosen when setting up the device. Data collection was performed after daily calibration of devices carried out using the quality phantom, according to the manufacturer’s instructions. Mean values of the right and left measurements were used for analyses, although both SI and T-score measurements had essentially the same distributions on the left and right foot across all 10 regions (Supplementary Figure S1).

### 2.3. Questionnaire Survey and Other Data Collection

Information on demographic, socio-economic, and lifestyle factors (including smoking, alcohol consumption, diet, and physical activity) and family and personal disease history was collected during the questionnaire survey [7,11]. Total physical activity was calculated through combining the activities (in metabolic equivalent of tasks [MET]-hours) from occupation, commuting, household activities, and leisure time exercises [15]. Standing height was measured to the nearest 0.1 cm using a portable stadiometer without shoes. Leg length was calculated as the difference between standing height and sitting height. Weight was measured to the nearest 0.1 kg using the TBF-300 Body Composition Analyzer (Tanita Inc., Tokyo, Japan), and BMI was calculated as weight in kilograms divided by height in meters squared. In addition, TBF-300 used foot-to-foot bioelectrical impedance analysis to measure body fat percentage (BF%) using its built-in proprietary algorithm [16]. Hand grip strength was measured using a hydraulic hand dynamometer (Jamar J00105, Jafayette Instrument Company, Inc., Jafayette, IN, USA) while participants were asked to sit upright in a chair, place their forearms on armrests, and squeeze the handle of the dynamometer as strongly as possible for three seconds [17]. For each participant, one measurement was obtained from each hand and the average of the two measurements was used for this analysis.

### 2.4. Follow-Up and Outcome Measures

The vital status of each participant was determined periodically through China’s Disease Surveillance Points (DSP) system (i.e., death registry), checked annually against local residential records and health insurance data and supplemented by annual active confirmation through street committees or village administrators, in order to minimise any loss to follow-up [13]. In addition, further information about incidence of major diseases (i.e., ischemic heart disease, stroke, cancer, and diabetes) and episodes of any hospitalisation were collected through electronic linkage with disease registries and nationwide health insurance claim databases (which has almost universal, ~99%, coverage for CKB participants). We used each participant’s unique national identification number as identifier to ensure a correct linkage with clinical records. All deaths and hospital admissions (including osteoporosis and fracture) were coded using the International Classification of Diseases, 10th Revision (ICD-10), by trained clinical staff who had no access to other information collected in the CKB study. By 1 January 2019, only 3498 (0.7% of ~0.5 million baseline participants) were lost to follow-up.

For the present analysis, we defined fracture-prone individuals using ICD-10 codes S02-S92 and M80-82, which cover any fracture and osteoporosis [18]. Only the first hospitalisation of fracture or osteoporosis during the follow-up period was considered and participants contributed person-years from time of resurvey (i.e., the time of BMD measurement) until the date of fracture/osteoporosis incidence, loss to follow-up, or global censoring date (i.e., 1 January 2019).

### 2.5. Statistical Analyses

Among the 25,239 CKB participants who attended the resurvey, calcaneus QUS data were available among 24,780 men and women. After excluding 129 participants who had missing questionnaire information, 24,651 were included in the current analysis. Means (standard deviations, SDs) or percentages of population characteristics were calculated overall and by men and women separately while adjusting for age and region where appropriate, using either multiple linear (for continuous outcomes) or logistic (for binary outcomes) regression.

Using the same formula (i.e., BMD = 0.002592 × [BUA + SOS] − 3.687) as used in the UK Biobank [19], we estimated BMD for each individual and calculated the Spearman correlation between eBMD and SI provided by the Achilles EXP II (GE Healthcare, Boston, MA, USA). Multiple linear regression analyses were conducted to calculate the least-squares means of SI and T-score for men and women separately in each 5-year age group, in each region and across different groups of age-at-menopause, adjusting for region and age, where appropriate. Similarly, least squares means of these bone health parameters were calculated in men and women by categories of socio-economic and lifestyle factors including education, income, smoking, alcohol consumption, physical activity, and tea consumption, adjusting for age, region, and other factors. Cross-sectional associations of various anthropometric parameters with BMD measurements in men and women separately were also assessed using multiple linear regression adjusting for the covariates mentioned above, plus menopause status in women. Heterogeneity of regression coefficients between men and women was examined using the Z-test. Cox proportional hazards models were used to estimate hazard ratios (HRs) of incident fracture related to each BMD parameter in men and women. Models were stratified by age at risk and region, and they were adjusted for age at QUS measurement; education; income; smoking; physical activity; consumption of alcohol, tea, fresh fruit, wholegrain, and milk, and, in women, menopause status. The proportional hazards assumption was assessed by use of Schoenfeld residual tests, and the results showed no evidence of violation of this assumption. We used group-specific variances to estimate the 95% confidence intervals (CI) for all categories, enabling comparisons between any two categories and not only with the reference group [20]. Each participant’s follow-up time was calculated as the time interval between QUS measurement and death, loss to follow-up, incident fracture, or global censoring date, whichever came first. Heterogeneity of HRs between men and women was estimated using the chi-squared test.

All analyses were conducted using SAS (version 9.3). Graphs were plotted using R 3.3.2.

## 3. Results

Among the 24,651 participants included in the analysis, the overall mean (SD) age was 59.5 (10.1) years, 61.7% were women, and 43.1% came from urban areas (Table 1). More than half of the male participants were current smokers (50.9%), but only 1.6% of women currently smoked. Alcohol and tea drinking were also more prevalent in men than in women. The overall mean (SD) was 88.6 (16.6) for SI and −0.72 (1.12) for T-score. Although men had a much higher mean SI than women (92.8 vs. 86.0), mean T-score was lower in men than in women (−0.85 vs. −0.64). SI and eBMD were highly correlated with each other (Spearman correlation coefficient = 0.98, Supplementary Figure S2).

**Figure 1:**
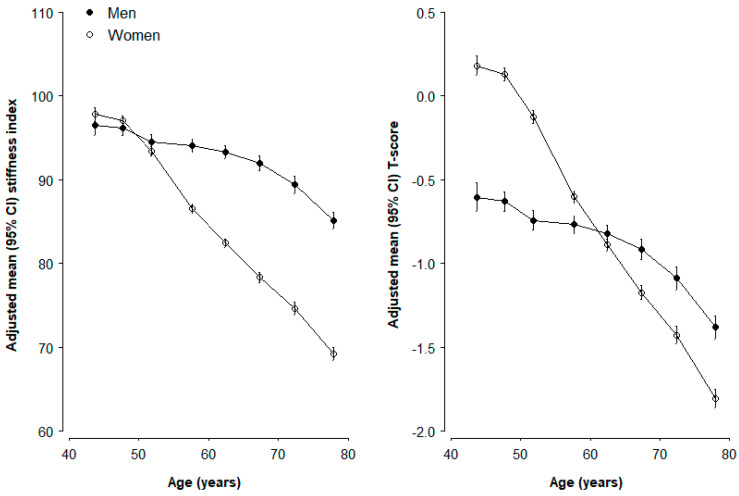
**Adjusted mean levels of stiffness index (SI) and T-score by age and sex.** Values were adjusted for region, and vertical lines crossing the dots represent 95% CIs. Solid black dots represent data from men, and open dots represent women.

SI and T-score both presented an inverse trend with age (Figure 1). For SI, it was similar in men and women before the age of 50 years, but after that, it dropped linearly and dramatically in women but only slowly in men. For women, SI at about 80 years old was about 25.8% lower when (93.6 vs. 69.5) than those younger than 50 years, corresponding to 10% decline per 10 year of age increase. In men, however, the rate of SI decline was much slower, from 94.6 at around 50 years old to 85.2 at around 80 years old (~3.8% per 10 year). At about 80 years old, men on average had about 18% higher SI than women (85.2 vs. 69.5, Figure 1). The age pattern of T-score was different from that for SI. In the young age group (i.e., before 60 years), women had a much higher T-score than men. This gender difference became smaller with age and disappeared at age 60 years, and it gradually increased after that, but with men having higher T-score than women (Figure 1).

SI was consistently higher in men than in women across all 10 regions, although large regional variations were observed for mean SI in both men and women (Supplementary Figure S3a). Overall, people from urban areas had higher SI than those from rural areas (90.6 vs. 87.1). For both men and women, those from Suzhou (an urban area near Shanghai) had the highest SI (97.1/89.8 in men/women), while men in Henan and women from Sichuan (both are rural regions) had the lowest values (89.5 and 82.2, respectively). However, for T-score, although the regional variation pattern was similar to that for SI, the gender difference was in an opposite direction, with men having a lower T-score than women across all 10 regions (Supplementary Figure S3b).

As expected, post-menopausal women on average had lower SI than men and pre-menopausal women (82.5 vs. 93.7 and 90.6, respectively, Supplementary Figure S4a). In addition, women who experienced menopause at a younger age had slightly lower SI than those who had late menopause. However, for T-score, surprisingly the mean value in men (−0.81) was even slightly lower than the average level of post-menopausal women (−0.76), much lower than that in premenopausal women (−0.32) (Supplementary Figure S4b).

Despite the above-described differences in SI and T-score, they had nearly identical associations with socio-economic and lifestyle factors (Table 2). Longer education (in women) and higher household income (in men) tended to be associated with better bone health (i.e., higher SI and T-score), although the multi-adjusted associations were not statistically significant. Never smoking (in men), regular tea drinking (in women), and higher physical activity were related to better bone health (*P*_trend_ < 0.001 for all). For instance, male current smokers had 2.1-unit lower SI and 0.14-unit lower T-score than in male never smokers. The corresponding differences in women were only about half, 1.2 and 0.08 units, respectively (*P*_trend_ = 0.18 for SI and 0.16 for T-score). These differences were quantitatively similar to those between female regular and never tea drinkers, despite the opposite directions, i.e., regular tea drinking was related to 1.2-unit higher SI and 0.07-unit higher T-score in contrast to never tea drinkers (*P*_trend_ = 0.0003 for both). Milk consumption was not associated with bone health in men, but in women, it was inversely related to both SI and T-score, with regular milk consumers having 1.2-unit lower SI and 0.09-unit lower T-score than never consumers (*P*_trend_ < 0.001). Higher consumption of fresh fruit and coarse grains was associated with higher SI and T-score in both men and women (*P*_trend_ < 0.05), but no clear associations were found of other dietary factors (including use of nutrient supplements) and vitamin D level measured at baseline with either SI or T-score.

**Figure 2:**
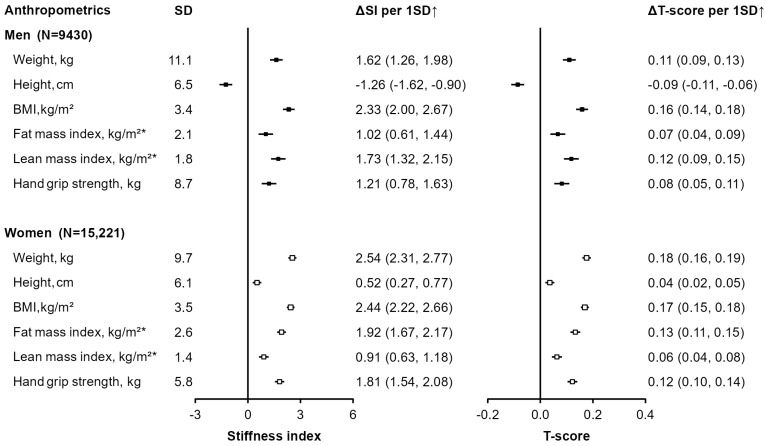
**Associations of anthropometric measures with stiffness index (SI) and T-score.** All analyses were adjusted for age; region; education; income; smoking; physical activity; consumption of alcohol, tea, fresh fruit, milk, and coarse grains; and, in women, menopause status. * Analyses were mutually adjusted for fat mass index and lean mass index. Black squares represent values in men and white squares represent values in women. Horizontal lines crossing the point estimates represent 95% CIs. *p* values for heterogeneity between men and women were <0.05 for all except for BMI, which was 0.60 for SI and 0.45 for T-score. SD↑ indicates per standard deviation higher.

Among the anthropometric measures studied, BMI was also the strongest correlate of bone health in men (per SD higher BMI was associated with 2.33-unit higher SI and 0.16-unit higher T-score), but in women, body weight had a slightly stronger association with SI and T-score (2.54 and 0.18 unit, respectively) than BMI (2.44 and 0.17 unit, respectively) (Figure 2). In men, lean mass had >70% stronger association with bone health parameters than fat mass (i.e., 1.73 vs. 1.02 SI and 0.12 vs. 0.07 T-score), but in women, the opposite was true, with fat mass having over two times stronger associations with SI (1.92 vs. 0.91) and T-score (0.13 vs. 0.06) than lean mass. Height was weakly but positively associated with SI and T-score in women, but in men, a much stronger but inverse association was observed, with each SD taller height, ~6.5 cm, associated with 1.26-unit lower SI and 0.09-unit lower T-score.

**Figure 3:**
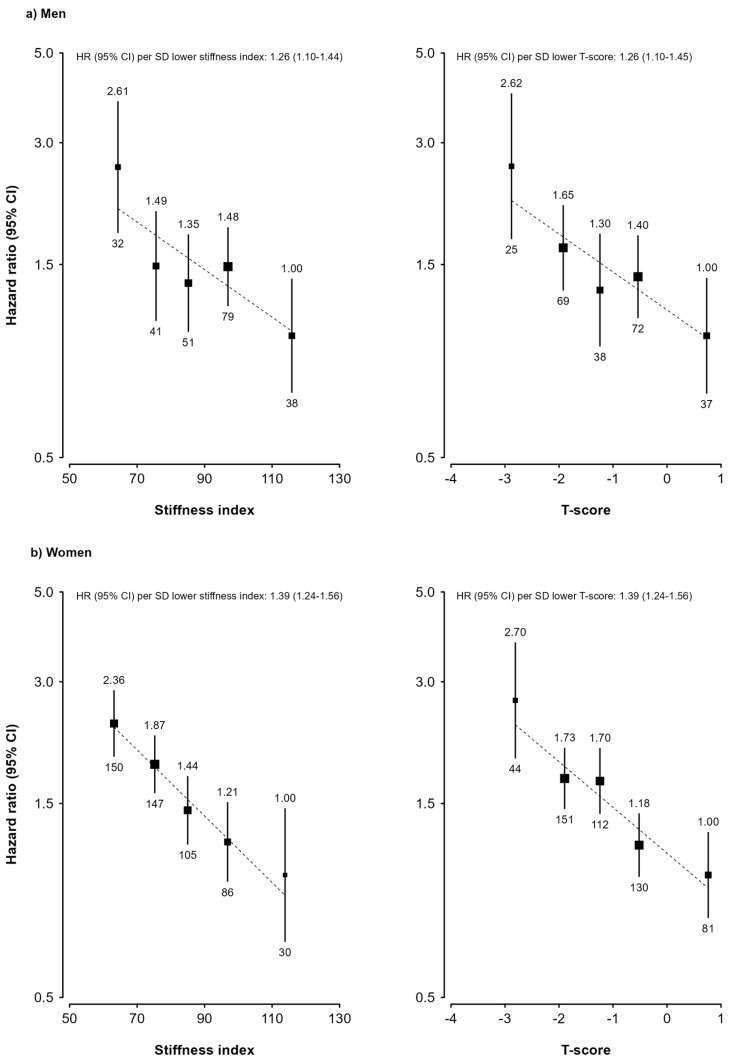
**Hazard ratios of stiffness index and T-score with risk of fracture in the China Kadoorie Biobank.** Cox regression models were stratified by age-at-risk and region and adjusted for age; education; income; smoking; physical activity; consumption of alcohol, tea, fresh fruit, coarse grains, and milk; and, in women, menopause status. Vertical lines crossing the black squares (point estimates of HRs) represent 95% CIs of HRs. The numbers above the vertical lines are the HRs, and the numbers below the vertical lines are the number of cases. The dashed lines represent linear associations of either SI or T-score with risk of fracture. Panel (**a**) represents results for men, and panel (**b**) represents results for women. *p* values for heterogeneity between men and women was 0.26 for SI and 0.27 for T-score.

Both SI and T-score were significantly and inversely associated with fracture risk in both men and women, and these associations were in a log-linear manner, with no clear threshold identified at which fracture risk increased (Figure 3). The associations were slightly but non-significantly stronger in women than in men, and with each SD lower SI, the risk of fracture was 26% (95% confidence interval: 10–44%) higher in men and 39% (24–56%) higher in women. T-score had essentially identical linear association with fracture in both men (HR 1.26, 1.10–1.45) and women (HR 1.39, 1.24–1.56). Comparing to those with T-score higher than 0, men with T-score lower than −2.5 had a HR of 2.62 (95%CI: 1.73–3.97), and the corresponding HR in women was 2.70 (95%CI: 1.94–3.75).

## 4. Discussion

In this relatively large study of Chinese adults from both rural and urban areas, we found that BMD measures assessed using QUS, i.e., SI and T-score, declined with age, particularly in women and from 50 years old. As expected, early menopause, smoking, and low physical activity were associated with lower BMD, while regular tea drinking, regular consumption of fresh fruit and coarse grains, and higher BMI and body weight were related to higher BMD. Although SI was higher in men than in women, the gender difference of T-scores provided by the Lunar Achilles EPXII was in the opposite direction, particularly in those younger than 60 years old. Despite that, T-score and SI had essentially a same association with risk of subsequent fracture incidence, with each SD lower SI or T-score being associated with 26% (95% CI: 10–44%) higher risk in men and 40% (25–57%) or 39% (24–56%), respectively, higher risk in women.

Previous evidence from various populations generally supports a greater BMD in men than in women throughout adulthood. For instance, in the US National Health and Nutrition Examination Survey (NHANES) involving > 13,000 participants aged 20 years and older, BMD was significantly higher in men than in women in every age group, at all skeletal sites, and in every race/ethnic group [21]. In another study by Kruger et al. involving nearly 800,000 Asian adults over 21 years old (that did not include participants from mainland China), SI measured using calcaneal ultrasound (GE-Lunar Achilles) was consistently higher in men than in women, although the differences in 40–50-year-old groups were much smaller [22]. This was similar to what we have observed in our study. However, for T-score, a different age- and sex-pattern (from that of SI) was observed in our study and the study by Kruger et al., showing that the T-score of women was higher than that of men in those younger than 60 years old. Although the International Society for Clinical Densitometry (ISCD) recommends using data from a group of young (i.e., 25–30 years old) white women as a reference for calculating the T-scores for both women and men [23], the GE Achilles EXPII calculates T-score using imbedded values of population- and gender-specific young adults as references. To further confirm that the lower T-score in men than in women in our study, despite a higher SI, was indeed caused by a higher reference database used for male participants, we calculated the a new T-score using the same mean and SD (i.e., 97.5 and 14.1, respectively) values of SI (i.e., derived from female participants younger than 50 years old in the current study) and found that the new T-score was much higher in men than in women and its age- and sex-pattern was very similar to that for SI (Supplementary Figure S5). Although QUS-measured T-score should not be used for the diagnosis of osteoporosis, based on our findings from this current analysis, the T-score provided by GE Lunar Achilles EXPII for Chinese male adults (as well as men of other ethnic groups including Japanese, German, French, and Italian) may have been significantly underestimated and could not be directly compared with the T-score in women. Use of a uniform Caucasian female referent database for T-score calculation in men [24], as is the case for the Hologic Sahara bone sonometer used by the UK Biobank [25], would facilitate the comparison of osteoporosis burden in different genders and across different countries.

Previous evidence supports using calcaneal QUS measures to predict fracture risk [26,27,28]. For instance, in a most recent meta-analysis of nine prospective studies of over 46,000 men and women (mean age 70 years) from Asia, Europe, and North America, both BUA and SOS were significantly and inversely associated with risk of osteoporotic fracture, with HR per 1 SD lower QUS measure being about 1.4, similar to what we found in our prospective analysis associating SI and T-score with risk of total fracture [28]. These findings support the utility of QUS in large-scale population-based epidemiological studies, particularly those conducted in resource-limited areas, given the undeniable advantages of using QUS over an X-ray-based approach to measure BMD (e.g., radiation-free, portability of the device, no need of a registered X-ray technologist to operate and cost effectiveness).

Our findings are in a general agreement with existing literature, supporting the relevance of a healthy lifestyle (i.e., smoking, physical activity, and healthy diet) for bone health. Smoking has long been recognised as a major lifestyle risk factor for poor bone health as it affects the metabolism of hormones, body weight, and antioxidant absorption, as well as increasing oxidative stress, thus disrupting healthy bone resorption and formation [29]. In contrast, previous findings on alcohol consumption and BMD were less consistent, with some showing a U-shaped association [30,31] and others showing no association [32], including our current study. There is also a lack of consistency in terms of tea consumption with BMD and fracture risk in the existing literature [33]. However, in the current study, we observed a clear positive association of tea consumption with SI and T-score (although only borderline significant in men), consistent with few previous reports in China [34] and other populations [35]. Several plausible mechanisms have been proposed, including biologically active compounds in tea, such as polyphenols (or catechins), fluoride, chromium, antioxidants, and phytoestrogens, which are biologically active and can improve bone biology and BMD by enhancing osteoblastic proliferation and differentiation, as well as promoting osteoclast apoptosis [36]. Previous evidence showed that either strenuous exercise, mild exercise, or weight-bearing activities could promote bone formation and bone mineral apposition, thus increasing BMD [37]. Our findings support this, but for the next step, detailed analysis is warranted to better understand the exact relevance of different types of physical activity to bone health and fracture risk. Among the dietary factors investigated, only fresh fruit and coarse grain were positively associated with BMD, while milk consumption was related to a significantly lower BMD in women. Fruit and coarse grain are the two major food groups contributing to the intake of dietary fibre and antioxidants in our study population [38,39]. Although not all consistent, several previous epidemiological studies have reported the potential relevance of fruit and fibre consumption for better bone health [40,41,42], and that the nutritional acid load [43], antioxidant vitamins [44,45] and phytochemicals [46], and gut microbiome [47] may all be involved in the underlying pathways. However, for the latter association, given the cross-sectional nature of the study, reverse causality, i.e., women with a known status of poor BMD potentially having increased their milk intake in order to improve bone health, might have been the main cause of the unexpected result.

Low BMI is a well-established risk factor for low BMD and fracture, particularly hip fracture in adult men and women. Our results on anthropometrics, including adiposity measures and hand grip strength, in relation to BMD measures, are broadly supporting of this [48]. However, there is still a lack of consistency in the literature regarding the relative contributions of lean and fat mass to BMD [49]. The gender-dependent patterns of the body composition–bone relationship observed in this study (i.e., fat mass was more strongly associated with BMD than lean mass in women, while the opposite was observed in men) was in broad agreement with some, but not all, previous reports [50,51]. Although the positive association between adiposity and BMD should not be utilised to challenge public health recommendations to reduce obesity, given its many other undesirable health impacts, a better understanding of the exact pathways linking body fat mass and BMD would greatly benefit global strategies for prevention of osteoporosis and fracture. This is the same for height, which was found to have a significant inverse association with bone health in men, but not in women, in our study. This was consistent with the findings from a study involving 2000 adults in Australia [52], 8000 adolescents in China [53], and 400 peripubertal children in Japan [54]. More recently, Mendelian randomisation analyses of data from the UK Biobank have found a similar inverse association between height and BMD [55]. Together, these results suggest a critical role of sex hormones and growth factors in bone mass acquisition, accumulation, and loss.

The strength of this study is its large sample size and the availability of a wide range of lifestyle and anthropometric variables as well as data on subsequent fracture incidence, allowing us to examine the value of BMD measures in predicting fracture risk. However, this study has several limitations too. Firstly, participants who attended this resurvey might have included a large proportion of healthy volunteers because those who were severely handicapped may not have been able to travel to the survey clinics. Therefore, the prevalence of possible osteoporosis (i.e., T-score < −2.5) in our study might be much lower than the actual prevalence in Chinese adults. But this should not bias the observed associations. Secondly, we only had QUS measurement at one peripheral body site (calcaneus), and therefore the SI and T-score observed in the current study may not reflect BMD at other body sites or of the whole body. Previous studies, however, have found that QUS measures were well correlated with DXA-measured BMD in multiple body sites (e.g., femur neck and lumbar spine) [6], supporting the validity of QUS measures as BMD markers in association studies. Thirdly, the fracture and osteoporosis events in our follow-up data were identified using electronic health records of hospitalisations (i.e., from mortality and morbidity registries as well as health insurance databases), and no separate validation study has been conducted to examine the accuracy of these clinical diagnoses. However, results from a previous CKB publication support the quality of our follow-up data on fracture and osteoporosis [18]. Moreover, potential misclassification in analysis outcome would most possibly be non-differential, and therefore the observed relative risks (HRs) would not be biased [56]. Finally, as mentioned above, the cross-sectional nature of the analysis excludes the possibility of confirming the direction of associations of lifestyle and anthropometric factors with BMD.

## 5. Conclusions

In summary, our findings suggest people (particularly women who experienced early menopause) of advanced age with lower BMI may have a lower BMD. QUS measures could predict fracture risk, although the absolute T-score was comparable between men and women because of the gender-specific reference databases used. It could be a practical tool for monitoring bone health in resource-limited settings. Several lifestyle factors (such as smoking, physical activity, tea drinking, and fresh fruit and coarse grain consumption) and anthropometrics (e.g., BMI) might be involved in the pathogenesis of osteoporosis, but future studies are necessary to determine the causality of these relationships.

## Figures and Tables

**Table 1:** Participant characteristics by gender (n = 24,651).

	Men (n = 9430)	Women (n = 15,221)	Overall (n = 24,651)
Age, years	60.2 (10.3)	59.0 (10.0)	59.5 (10.1)
Urban, %	43.3	42.9	43.1
Education > 6 years, %	57.5	41.6	47.7
Annual household income ≥75,000 yuan	28.0	23.7	25.3
Current smoking, %	50.9	1.6	20.5
Current alcohol drinking, %	28.6	1.9	12.1
Regular tea drinking, % *	46.2	17.6	28.6
Physical activity, MET-hours/day	19.0 (15.2)	17.6 (12.7)	18.1 (13.7)
Regular dietary consumption, % ^†^			
Fresh fruit	39.0	48.7	45.0
Coarse grain	19.2	19.1	19.1
Milk	10.5	11.5	11.1
Red meat	60.4	52.4	55.5
Fish and shellfish	11.5	9.5	10.3
Eggs	41.4	38.1	39.4
Menopause, %	-	76.9	-
Menopause age, years	-	48.8 (4.5)	-
Supplement intake, %			
Calcium, iron, and zinc	14.5	21.6	18.9
Fish oil and cod fish oil	7.0	9.8	8.7
Multi-vitamins	6.4	8.9	8.0
25-Hydroxyvitamin D, ng/mL ^‡^	37.3 (10.3)	31.2 (8.5)	33.9 (9.8)
Body composition measures			
BMI, kg/m^2^	24.0 (3.4)	24.3 (3.5)	24.2 (3.5)
Standing height, cm	164.7 (6.5)	153.5 (6.1)	157.8 (8.3)
Body weight, kg	65.2 (11.2)	57.4 (9.7)	60.4 (11.0)
Fat body mass, kg ^§^	13.4 (5.9)	18.1 (6.5)	16.3 (6.7)
Fat mass index, kg/m^2 §^	4.9 (2.1)	7.6 (2.6)	6.6 (2.8)
Lean body mass, kg ^§^	51.9 (6.9)	39.3 (4.4)	44.1 (8.2)
Lean mass index, kg/m^2 §^	19.1 (1.8)	16.7 (1.4)	17.6 (2.0)
Hand grip strength, kg ^¶^	32.2 (8.7)	20.1 (5.8)	24.7 (9.2)
QUS parameters			
BUA, dB/MHz	113.3 (11.4)	107.5 (12.4)	109.7 (12.4)
SOS, m/s	1562.2 (37.2)	1551.6 (35.9)	1555.6 (36.8)
Stiffness index (SI)	92.8 (16.2)	86.0 (16.2)	88.6 (16.6)
T-score	−0.85 (1.11)	−0.64 (1.13)	−0.72 (1.12)
eBMD, g/cm^2^	0.66 (0.12)	0.62 (0.11)	0.64 (0.12)

Values are percentage or mean (SD) and were adjusted for age and region. BMI: Body Mass Index; BUA: Broadband Ultrasound Attenuation; eBMD: Estimated Bone Mineral Density, calculated using the formula from the UK Biobank: eBMD = 0.002592 × (BUA + SOS) − 3.687 [19]; MET: Metabolic Equivalent of Task; QUS: Quantitative Ultrasound; SOS: Speed of Sound. * Regular tea drinking was defined here as consuming tea at least once per week. ^†^ Regular dietary consumption was defined here as consuming these food groups ≥ 4 days per week. ^‡^ Measured using plasma samples collected at the CKB baseline survey and was available in 1677 participants. 1 nmol/L = 2.5 ng/mL. ^§^ 318 participants had missing values. ^¶^ 34 participants had missing values.

**Table 2:** Socio-economic and lifestyle factors in relation to stiffness index and T-score in men and women.

	Men (N = 9430)			Women (N = 15,221)		
	N	Stiffness Index	T-Score	N	Stiffness Index	T-Score
**Education**						
No formal education	853	92.0 (90.8, 93.1)	−0.91 (−0.99, −0.84)	4107	85.7 (85.2, 86.1)	−0.66 (−0.70, −0.63)
Primary school	3157	93.5 (92.9, 94.1)	−0.81 (−0.85, −0.77)	4782	86.0 (85.6, 86.4)	−0.64 (−0.67, −0.61)
>6 years	5420	92.5 (92.1, 93.0)	−0.87 (−0.91, −0.84)	6332	86.2 (85.8, 86.6)	−0.63 (−0.65, −0.60)
*P*_trend_		0.63	0.68		0.14	0.15
**Annual household income, yuan/year**						
<35,000	3515	92.2 (91.6, 92.8)	−0.90 (−0.94, −0.86)	6406	86.2 (85.8, 86.5)	−0.63 (−0.65, −0.60)
35,000–74,999	3270	93.2 (92.6, 93.7)	−0.83 (−0.87, −0.79)	5212	86.1 (85.8, 86.5)	−0.63 (−0.66, −0.60)
≥75,000	2645	93.2 (92.5, 93.8)	−0.83 (−0.88, −0.79)	3603	85.4 (84.9, 85.9)	−0.68 (−0.71, −0.64)
*P*_trend_		0.05	0.05		0.05	0.05
**Smoking**						
Never regular	3136	93.9 (93.3, 94.4)	−0.78 (−0.82, −0.74)	14,890	86.0 (85.8, 86.2)	−0.64 (−0.65, −0.62)
Ex-regular	1491	93.7 (92.9, 94.5)	−0.79 (−0.85, −0.74)	93	85.4 (82.6, 88.2)	−0.69 (−0.88, −0.50)
Current regular	4803	91.8 (91.4, 92.3)	−0.92 (−0.95, −0.89)	238	84.8 (83.1, 86.6)	−0.72 (−0.85, −0.60)
*P*_trend_		<0.001	<0.001		0.18	0.16
**Alcohol drinking**						
Never regular	5775	92.7 (92.3, 93.1)	−0.86 (−0.89, −0.84)	14,738	86.0 (85.8, 86.2)	−0.64 (−0.65, −0.62)
Ex-regular	961	93.0 (91.9, 94.0)	−0.85 (−0.91, −0.78)	192	85.4 (83.5, 87.3)	−0.68 (−0.82, −0.55)
Current regular	2694	93.1 (92.4, 93.7)	−0.84 (−0.88, −0.80)	291	85.4 (83.9, 87.0)	−0.68 (−0.79, −0.57)
*P*_trend_		0.30	0.31		0.39	0.37
**Tea drinking**						
Never	3191	92.3 (91.7, 92.9)	−0.89 (−0.93, −0.85)	9398	85.6 (85.4, 85.9)	−0.66 (−0.68, −0.64)
Occasional	1878	93.1 (92.3, 93.8)	−0.84 (−0.89, −0.79)	3143	86.3 (85.8, 86.8)	−0.62 (−0.65, −0.58)
Regular	4361	93.1 (92.6, 93.6)	−0.83 (−0.87, −0.80)	2680	86.8 (86.2, 87.3)	−0.59 (−0.62, −0.55)
*P*_trend_		0.06	0.05		<0.001	<0.001
**Physical activity ^†^**						
1st tertile	3108	91.8 (91.3, 92.4)	−0.92 (−0.96, −0.88)	5072	85.2 (84.8, 85.6)	−0.69 (−0.72, −0.67)
2nd tertile	3180	92.9 (92.4, 93.5)	−0.85 (−0.89, −0.81)	5076	86.2 (85.8, 86.6)	−0.63 (−0.65, −0.60)
3rd tertile	3142	93.7 (93.1, 94.3)	−0.80 (−0.84, −0.76)	5073	86.5 (86.1, 86.9)	−0.60 (−0.63, −0.58)
*P*_trend_		<0.001	<0.001		<0.001	<0.001
**Dietary Consumption**						
**Fresh fruit**						
Less than weekly	2483	92.0 (91.3, 92.6)	−0.91 (−0.95, −0.87)	3043	85.4 (84.9, 85.9)	−0.68 (−0.71, −0.64)
Weekly	4231	93.2 (92.7, 93.6)	−0.83 (−0.87, −0.80)	6454	86.0 (85.7, 86.4)	−0.64 (−0.66, −0.61)
Daily	2716	93.0 (92.4, 93.7)	−0.84 (−0.88, −0.80)	5724	86.2 (85.9, 86.6)	−0.62 (−0.65, −0.60)
*P*_trend_		0.03	0.03		0.02	0.02
**Coarse grain**						
Never/rarely	3955	92.3 (91.8, 92.9)	−0.89 (−0.93, −0.85)	5614	85.6 (85.2, 86.0)	−0.67 (−0.70, −0.64)
Monthly	1991	92.5 (91.8, 93.2)	−0.87 (−0.92, −0.83)	3483	86.0 (85.5, 86.5)	−0.64 (−0.67, −0.61)
At least weekly	3484	93.5 (92.9, 94.2)	−0.81 (−0.85, −0.76)	6124	86.4 (86.0, 86.8)	−0.61 (−0.64, −0.59)
*P*_trend_		0.02	0.02		0.01	0.01
**Milk**						
Never/rarely	7073	92.7 (92.3, 93.1)	−0.86 (−0.89, −0.84)	11,377	86.2 (86.0, 86.5)	−0.62 (−0.64, −0.61)
Monthly−3 days/week	1368	93.3 (92.4, 94.1)	−0.82 (−0.88, −0.77)	2096	85.5 (84.9, 86.1)	−0.67 (−0.71, −0.63)
>4 days/week	989	93.1 (92.1, 94.2)	−0.83 (−0.90, −0.76)	1748	85.0 (84.4, 85.7)	−0.71 (−0.75, −0.66)
*P*_trend_		0.24	0.24		<0.001	<0.001

Analyses were adjusted for age (continuous), region (10 regions), and all other variables listed in the table. ^†^ Sex-specific tertiles were defined using the following cut-off values: 9.40 and 22.20 MET-hours/day in women and 10.09 and 19.60 Met-hours/day in men.

## Data Availability

The China Kadoorie Biobank (CKB) is a global resource for the investigation of lifestyle, environmental, blood biochemical, and genetic factors as determinants of common diseases. The CKB study group is committed to making the cohort data available to the scientific community in China, the UK, and worldwide to advance knowledge about the causes, prevention, and treatment of disease. For detailed information on what data are currently available to open access users and how to apply for them, visit https://www.ckbiobank.org/data-access (accessed on 24 February 2025). Researchers who are interested in obtaining the raw data from the China Kadoorie Biobank study that underlines this paper should contact ckbaccess@ndph.ox.ac.uk. A research proposal will be requested to ensure that any analysis is performed by bona fide researchers and—where data are not currently available to open access researchers—is restricted to the topic covered in this paper.

## Supplementary Materials

The following supporting information can be downloaded at: https://www.mdpi.com/article/10.3390/nu17050865/s1, Table S1: Reference population data imbedded in the GE Achilles EXPII quantitative ultrasound (QUS) device; Figure S1: Distribution of stiffness index and T-score in right and left feet across 10 regions; Figure S2: Correlation between stiffness index and estimated bone mineral density; Figure S3: Adjusted mean levels of stiffness index (SI) (a) and T-score (b) across 10 regions; Figure S4: Adjusted mean levels of stiffness index (SI) (a) and T-score (b) by menopause status. Figure S5: Adjusted mean levels of re-calculated T-score using CKB young female participants’ SI as reference for both men and women.
