# Supplementary material for: Patterns and Correlates of Bone Mineral Density Parameters Measured Using Calcaneus Quantitative Ultrasound in Chinese Adults
Source: Nutrients. 2025 Feb 28;17(5):865. doi: 10.3390/nu17050865 (PMC11901691; doi:10.3390/nu17050865)
Supplement: Supplementary file 1 [file nutrients-17-00865-s001.zip › nutrients-3484667-supplementary.pdf]

Table S1. Reference population data imbedded in the GE Achilles EXPII quantitative ultrasound (QUS) device. *

	Subgroups					Subgroups			
	AF	AM	PF	PM		AF	AM	PF	PM
Japanese	×	×	×	×	Italian	×	×		
German	×	×			Middle East	×			
European	×				Chinese	×	×		
French	×				Greek	×			
Latin American	×	×			Saudi Arabian	×			
Mercosurian	×	×			USA	×			

AF: adult female; AM: adult male; PF: paediatric female; PM: paediatric male. * Provided by GE at https://www.gehealthcare.com/-/media/576a00f085a74a96a1b9af70e20ff197.pdf (accessed on 23/08/2019).

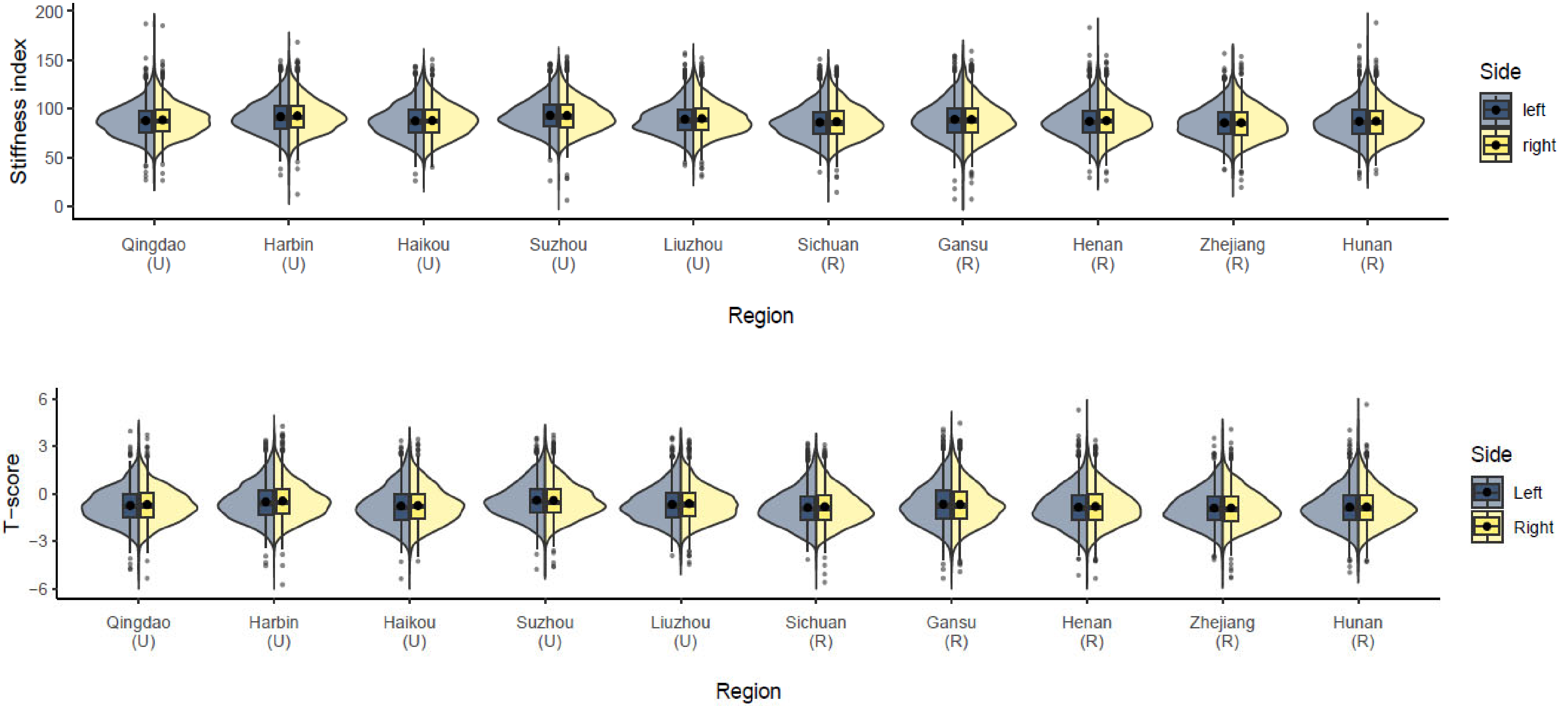

Figure S1. Distribution of stiffness index and T-score in right and left feet across 10 regions.

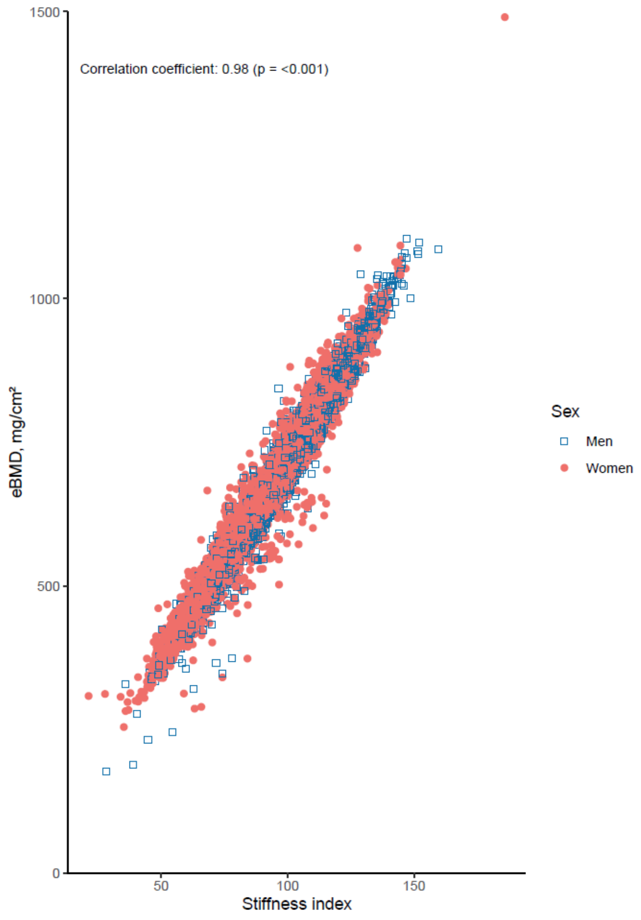

Figure S2. Correlation between stiffness index and estimated bone mineral density.

eBMD: Estimated Bone Mineral Density, calculated using the formula from the UK Biobank: eBMD = 0.002592 × (BUA + SOS) − 3.687

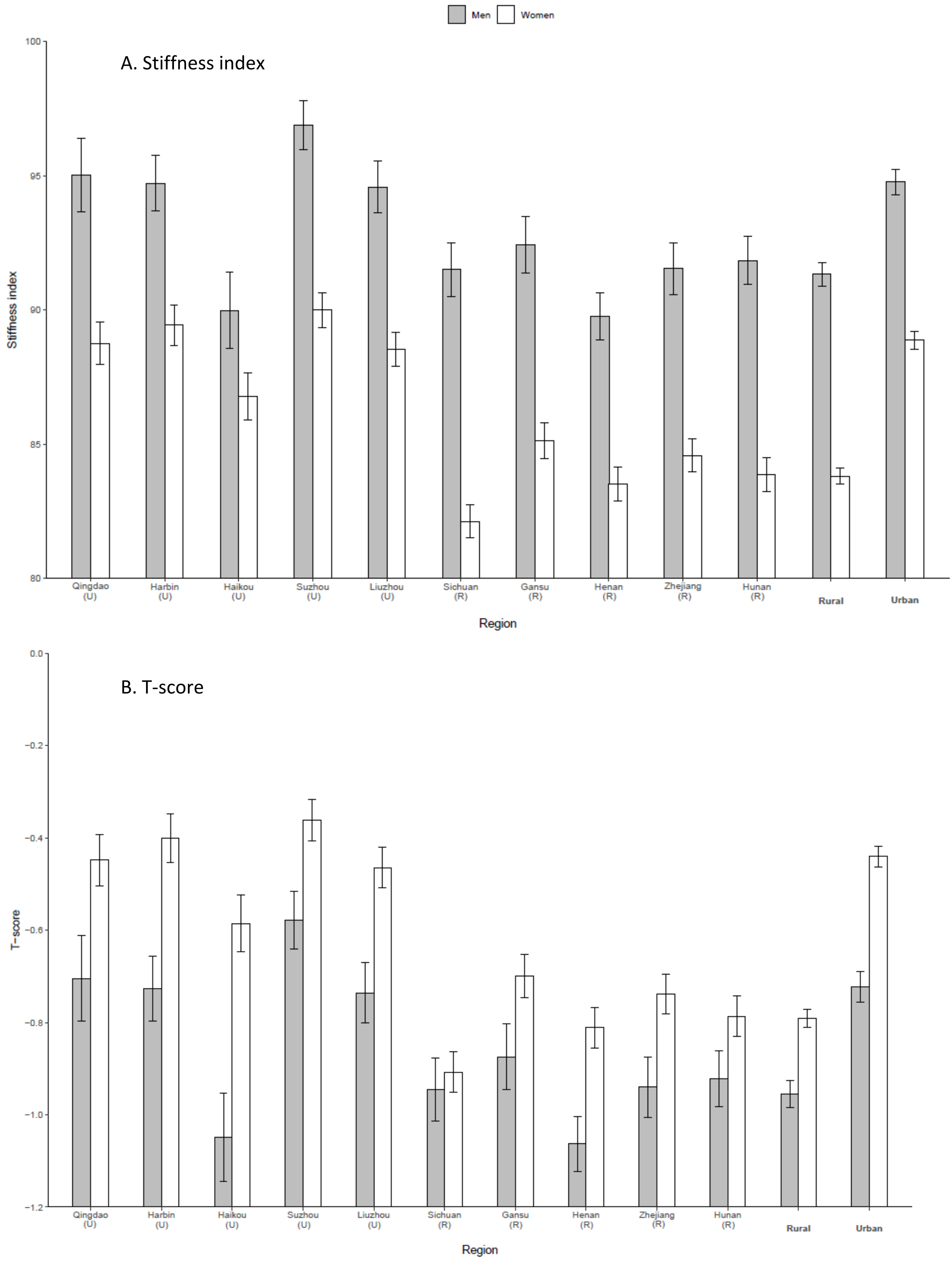

Figure S3. Adjusted mean levels of stiffness index (SI) (**A**) and T-score (**B**) across 10 regions. * Values were adjusted for age. Values were adjusted for age and vertical lines on top of the grey and white bars represent 95% CIs. Grey bars represent men and white bars represent data in women.

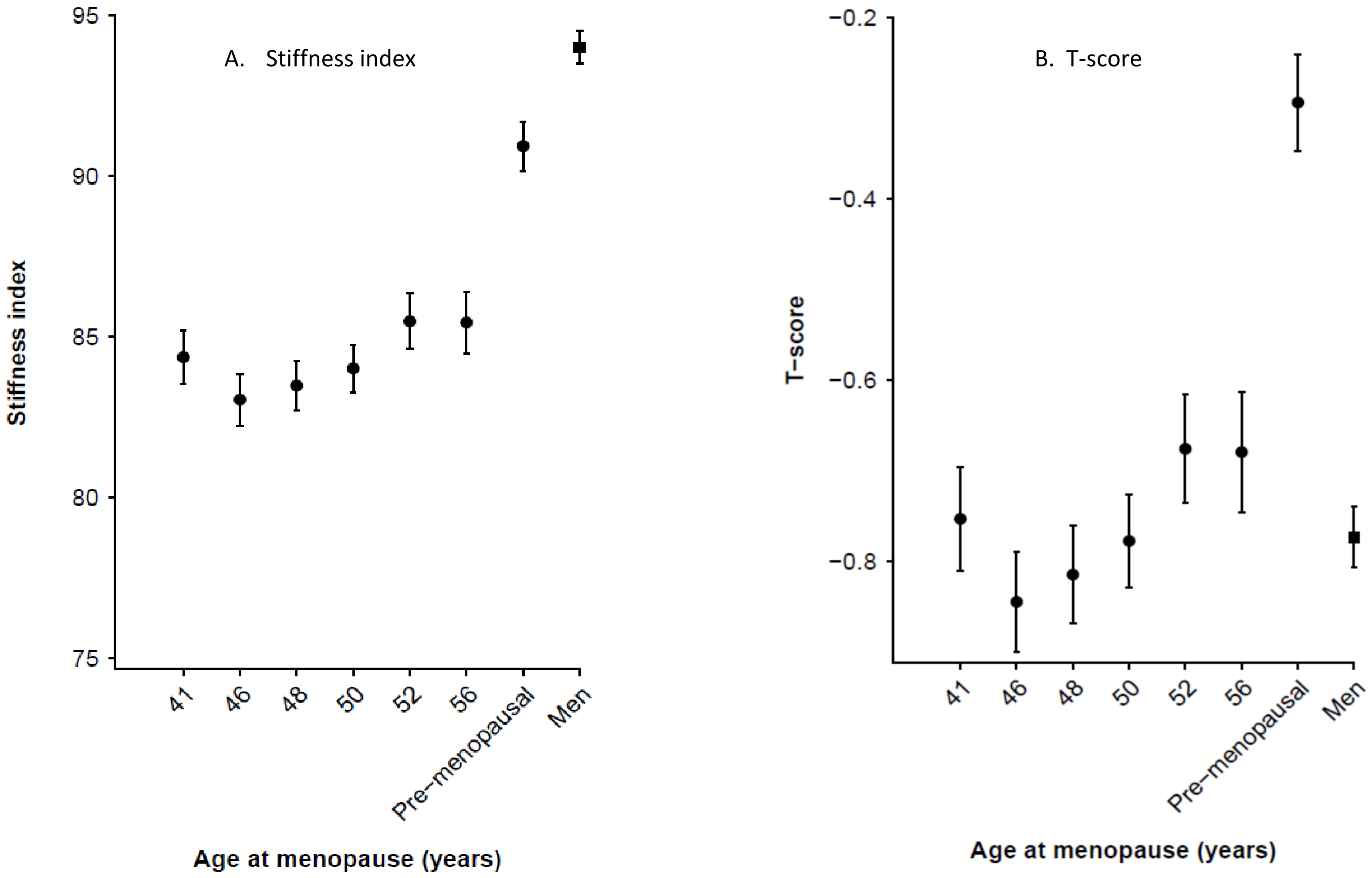

Figure S4. Adjusted mean levels of stiffness index (SI) (**A**) and T-score (**B**) by menopause status. * Values were adjusted for age, region, education, income, smoking, physical activity, and consumption of alcohol, tea, fresh fruit, coarse grains and milk. The black dots represent data from women and the black square represent the data from men. Vertical lines crossing these point estimates represent 95% CIs.

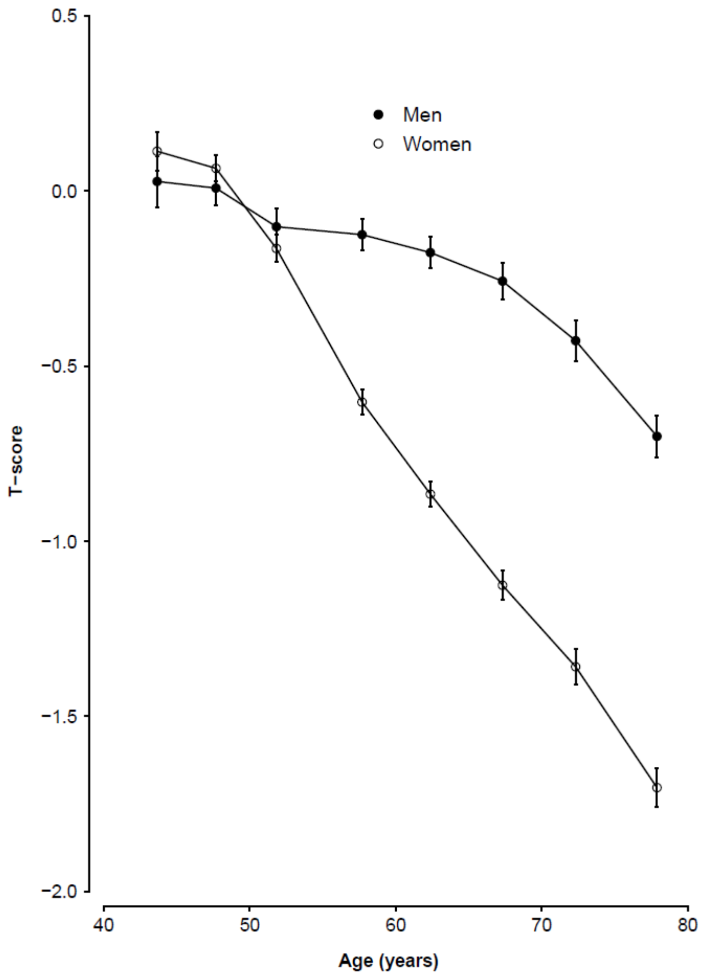

Figure S5. Adjusted mean levels of re-calculated T-score using CKB young female participants' SI as reference for both men and women. Values were adjusted for region.
